# Knowledge and attitudes toward clinical laboratory medicine among undergraduate medical interns in China: a cross-sectional survey

**DOI:** 10.3389/fmed.2025.1671631

**Published:** 2025-10-13

**Authors:** Yonggang Yang, Jiyun Tian, Baobing Chen, Song Chen

**Affiliations:** Department of Clinical Laboratory, Hangzhou Third People’s Hospital, Hangzhou, Zhejiang, China

**Keywords:** medical school curriculum, laboratory medicine knowledge, clinical medicine students, undergraduate medical education, medical interns

## Abstract

**Introduction:**

Undergraduate clinical medical interns often lack systematic laboratory medicine training, potentially impacting their diagnostic reasoning and patient safety. This study aimed to assess the perceived knowledge and attitudes toward clinical laboratory medicine among this population in China, addressing a significant gap in medical education evaluation.

**Methods:**

A cross-sectional study was conducted from March to April 2025 across 11 general hospitals in Eastern China (Shanghai, Hangzhou, Nanjing, Suzhou, Ningbo, Xuzhou, Shaoxing, Yangzhou, Huzhou, and Taizhou). The self-developed and validated 13-item Clinical Laboratory Knowledge and Attitudes Questionnaire (CLKAQ) was structured in three domains: Knowledge, Attitudes and Suggestions. All 303 clinical interns completed the instrument. SPSS 25.0 and AMOS 26.0 were used. Descriptive statistics (frequencies/percentages for qualitative data; mean ± SD for quantitative data) summarized characteristics, knowledge, and attitudes. Scale reliability and validity were confirmed. Normality was assessed via Kolmogorov–Smirnov test. Group comparisons (gender, age, city tier) employed Mann–Whitney U and Kruskal-Wallis tests. Spearman correlation examined knowledge-attitude relationships. Multiple response and content analysis supplemented quantitative findings.

**Results:**

The mean self-perceived knowledge scale score (5-point Likert scale) among the 303 interns was 2.22 ± 0.424. The mean attitude scale score (5-point Likert scale) was 4.05 ± 0.312. Significant differences emerged in key competencies: Gender disparities in report interpretation (Q3), perceived importance of laboratory knowledge (Q5), and learning motivation (Q7); Age-group variations in perceived knowledge adequacy (Q1), (Q5) and (Q7); Interns from third-tier cities demonstrated consistently higher self-perceived competence across all knowledge and attitude dimensions than those in tier-1/2 cities (*p* < 0.05). A weak positive correlation linked knowledge and attitude levels (*r* = 0.171, *p* < 0.05). Critical differences were noted in preferred learning channels (Q10) and perceived barriers (Q11). Regarding open-ended questions, all interns expressed the need for increased clinical laboratory knowledge training and provided specific suggestions for such training.

**Conclusion:**

Undergraduate clinical interns demonstrated suboptimal clinical laboratory knowledge but expressed highly positive attitudes toward learning. This underscores the critical need to enhance clinical laboratory training during clerkship. Implementing measures to improve knowledge is necessary. These findings inform curriculum optimization and educational strategy development for clinical continuing education.

## 1 Introduction

The Accreditation Council for Graduate Medical Education (ACGME) requires residents to demonstrate knowledge of established and evolving biomedical, clinical, epidemiological, and social-behavioral sciences—encompassing scientific inquiry—and apply this knowledge to patient care (1). Clinical residency education typically progresses through three stages: undergraduate medical education, clerkship, and standardized residency training. However, persistent challenges exist—undergraduate education (2), clerkship (3), and standardized training (4–6)—such as the need to improve interns’ diagnostic and therapeutic capabilities, and their ability to integrate and analyze laboratory results in the diagnostic process. A fundamental requirement for graduates is the ability to select appropriate clinical laboratory tests based on patient condition, safety, and cost-effectiveness, justify their selection, and interpret the results (7). Clinical laboratory testing is central to disease diagnosis and treatment. Within the current medical education system, clinical medical students receive minimal medical laboratory science coursework or lack systematic rotation training within the clinical laboratory department, resulting in insufficient laboratory knowledge. The assessment of clinical reasoning and medical knowledge is becoming increasingly crucial in clinical practice, primarily due to the persistent and harmful problem of diagnostic errors (8, 9). Global studies confirm systemic issues: Saffar et al. (10) reported 47.9% of Iranian students self-rated as ‘weak’ in test interpretation; Alosaimi (11) found only 18.3% of Saudi interns passed basic microbiology assessments; Twisha et al. (12) documented 15% inappropriate test utilization in Australian hospitals. Despite these challenges and laboratory medicine’s critical role (9, 13) no study has comprehensively assessed self-perceived knowledge-attitude discrepancies among Chinese clinical interns.

To address this gap, we conducted the Clinical Laboratory Knowledge and Attitudes Questionnaire (CLKAQ) instrument with three key advancements over prior research: First multidimensional assessment of knowledge and attitudes among Chinese clinical interns; Samples come from different city tiers and direct prioritization of training needs from 303 interns’ quantitative/qualitative feedback. This study aims to analyze undergraduate clinical medicine interns’ knowledge and attitudes regarding clinical laboratory medicine through a questionnaire survey. The findings will highlight the necessity of enhancing laboratory medicine education within undergraduate clinical training and provide a reference for further optimizing interns’ continuing education curricula, educational improvements, and strategy development.

## 2 Methods

### 2.1 Questionnaire design and validation

Data were collected using a cross-sectional survey supplemented with qualitative content analysis of open-ended responses. Surveys as tools are widely used in Health Professions’ Education (HPE) (14). The Clinical Laboratory Knowledge and Attitudes Questionnaire (CLKAQ), specifically developed and validated for this study targeting undergraduate clinical medicine interns (Questionnaire details are provided in Supplementary Material 1), was constructed based on: the Assessment Guidebook (8) detailing the ACGME use of milestones for resident assessment; the ACGME Common Program Requirements (Residency) (1); the overall objectives outlined in the Chinese Undergraduate Medical Education Standards—Standards for basic medical education in China (2022 Edition) (7); a review of existing literature (10, 15, 16); and findings derived from focus group discussions and pilot testing.

The questionnaire comprised four sections: Part I collected participants’ basic information; Part II assessed self-perceived clinical laboratory knowledge through four items (Q1-Q4) utilizing a 5-point Likert scale (“Strongly Disagree” to “Strongly Agree”), evaluating perceived adequacy of knowledge for clinical job requirements, clarity of position-specific expectations, ability to independently interpret common reports (e.g., CBC, biochemistry, coagulation), and implementation capability of the clinical laboratory critical value reporting system; Part III measured attitudes via three Likert-scale items (Q5-Q7) addressing perceived importance of laboratory knowledge, with four additional non-scale multiple-choice items (Q8-Q11) investigating preferred knowledge domains, learning motivations, knowledge acquisition channels, and barriers. The final section (Part IV) consisted of two open-ended items: one eliciting a binary response regarding educational enhancement needs, followed by an item requesting specific training suggestions.

A multidisciplinary expert panel was convened, comprising 10 specialists: 2 medical educators, 2 residency program directors, 4 clinical specialists, and 2 laboratory medicine specialists. A pilot test was conducted with 31 clinical interns (excluded from the main study), confirming item clarity and reliability (Cronbach’s *α* > 0.7 reliability threshold) (17, 18).

The CLKAQ was developed and administered electronically via the WeChat-based Questionnaire Star mini-program. A QR code linked to the survey was generated within the platform and disseminated simultaneously to all potential participants. Participants accessed the questionnaire by scanning the QR code using WeChat. This process yielded 324 submitted responses. After data screening excluding 21 invalid entries, 303 valid questionnaires were retained for analysis (dropout rate of 6.48%). The CLKAQ’s psychometric properties were assessed using data from all 303 participants, yielding the following metrics for reliability and validity.

The Cronbach’s *α* for the knowledge dimension was 0.905 and for the attitudes dimension was 0.803, both exceeding the conventional threshold of 0.7 for acceptable internal consistency (17, 18); The Intraclass Correlation Coefficients (ICC) indicated moderate reliability for both the knowledge (ICC = 0.705) and attitudes (ICC = 0.576) dimensions, based on conventional thresholds (19). These thresholds define reliability as poor (<0.5), moderate (0.5–0.75), good (0.75–0.90), or excellent (>0.90). The 95% confidence intervals for these estimates are provided in Table 1.

**Table 1:** Descriptive statistics, reliability, and EFA of the CLKAQ scales (*N* = 303).

Dimension	Item	Min	Max	Mean	SD	Reliability		EFA	
						Cronbach’s *α*	ICC	Factor 1 (Knowledge)	Factor 2 (Attitudes)
Knowledge	Q1	2	3	2.19	0.397	0.905	0.705	0.841	
	Q2	2	4	2.25	0.465			0.897	
	Q3	2	3	2.22	0.413			0.885	
	Q4	2	3	2.23	0.420			0.839	
	Mean			2.22	0.424				
Attitudes	Q5	4	5	4.10	0.299	0.803	0.576		0.752
	Q6	3	5	4.03	0.303				0.848
	Q7	3	5	4.03	0.334				0.868
	Mean			4.05	0.312				

CLKAQ = Clinical Laboratory Knowledge and Attitudes Questionnaire; SD = Standard Deviation; ICC = Intraclass Correlation Coefficient; EFA = Exploratory Factor Analysis; Factor loadings <0.40 are omitted for clarity. Scales were scored on a 5-point Likert scale (1 = Strongly Disagree, 5 = Strongly Agree); Overall model fit: Extraction Method: Principal Component Analysis, Rotation Method: Varimax, Cumulative Variance Explained: 79.357%, KMO measure: 0.783, Bartlett’s Test of Sphericity: *χ*^2^ = 1574.626, *p* < 0.001.

Content validity was assessed by a panel of 10 experts. For each item, the Content Validity Ratio (CVR) was computed. Based on the recalculated critical values by Wilson et al. (20)(which corrected anomalies in the original Lawshe’s table), the critical CVR value for a one-tailed test at *α* = 0.05 was 0.520. The Item-Content Validity Index (I-CVI) was also calculated for each item, for which a value of ≥0.78 was deemed acceptable according to established guidelines (21). As detailed in Supplementary Material 2, all items demonstrated excellent content validity: all I-CVI values ranged from 0.90 to 1.00, and all CVR values exceeded the critical threshold of 0.520 (range: 0.80 to 1.00). Finally, the Scale-Content Validity Index/Average (S-CVI/Ave) was 0.97, indicating excellent content validity at the scale level (21).

Exploratory Factor Analysis (EFA) was conducted to examine the underlying factor structure of the questionnaire. The suitability of the data for EFA was confirmed by a Kaiser-Meyer-Olkin (KMO) measure of 0.783, exceeding the recommended threshold of 0.70 for factorability (22), and a statistically significant Bartlett’s test of sphericity (*χ*^2^ = 1574.626, *p* < 0.001) (23). Principal Axis Factoring was selected as the extraction method, consistent with the goal of identifying latent constructs. The analysis yielded a clear two-factor solution, which cumulatively explained 79.4% of the total variance. All items demonstrated strong and salient loadings on their primary factors (range: 0.76 to 0.92; Table 1), with no significant cross-loadings (all<0.30). The pattern of loadings cleanly corresponded to the pre-defined Knowledge and Attitudes dimensions, resulting in a simple structure with factors that are statistically independent.

### 2.2 Dimension correlation analysis

Spearman’s correlation analysis revealed a statistically significant weak positive correlation between the knowledge and attitudes dimensions (*r* = 0.171, *p* < 0.05; Table 2).

**Table 2:** Dimension correlation analysis (Spearman’s Rho).

Dimension	Knowledge	Attitudes
Knowledge	1.000	
Attitudes	0.171	1.000

*N* = 303 for all correlations; *p*-value for knowledge-attitudes correlation = 0.003 (two-tailed); Correlation interpretation: r (Spearman’s rank correlation coefficient) = 0.171.

CFA analysis in AMOS 26.0 validated the questionnaire’s factor structure, with post-modification fit indices for all six established criteria: the chi-square to degrees of freedom ratio (χ^2^/df), Root Mean Square Error of Approximation (RMSEA), Goodness of Fit Index (GFI), Normed Fit Index (NFI), Comparative Fit Index (CFI) and Tucker-Lewis Index (TLI) demonstrating satisfactory two-factor convergent validity and model fit, as detailed in Table 3. The confirmatory factor analysis demonstrated an acceptable fit of the two-factor model to the data. Although the RMSEA value of 0.063 slightly exceeded the stringent cutoff of 0.06 proposed by Hu and Bentler (24), it is well within the ‘reasonable fit’ range (0.05–0.08) as recommended by MacCallum et al. (25). This, combined with the excellent performance of other key indices (CFI = 0.995, >0.95; TLI = 0.984, > 0.95; χ^2^/df = 2.214, < 3), provides strong evidence for the construct validity of the proposed two-factor structure.

**Table 3:** CFA fit indices for CLKAQ scales.

Model fit indices	*χ*^2^/df	RMSEA	GFI	NFI	CFI	TLI
Two-Factor Model	2.214	0.063	0.988	0.992	0.995	0.984
Threshold for Acceptance	<3	<0.06	>0.90	>0.90	>0.95	>0.95
Interpretation	Excellent	Good	Good	Good	Excellent	Excellent

Model fit was evaluated using multiple indices. The root mean square error of approximation (RMSEA) value of 0.063 indicates a reasonable fit according to the criteria proposed by MacCallum et al. (25), who suggest that values up to 0.08 represent a reasonable error of approximation in the population. Furthermore, the comparative fit index (CFI) and Tucker-Lewis index (TLI) both exceeded the recommended threshold of 0.95, and the chi-square/degrees of freedom ratio (χ^2^/df) was below 3. The collective pattern of these fit indices supports the acceptability of the two-factor model (24).

### 2.3 Sample

This cross-sectional online study assessed clinical laboratory knowledge and attitudes among undergraduate clinical medicine interns in China. Participants were recruited between March 28 and April 28, 2025, during the mid-to-late clerkship phase when interns had completed rotations across most hospital departments. The self-administered online questionnaire (CLKAQ) was distributed via the Questionnaire Star platform as a single cross-sectional survey. Eleven general hospitals representing varying economic development tiers (tier-1: Shanghai, Hangzhou, Nanjing; tier-2: Suzhou, Ningbo, Xuzhou, Shaoxing; tier-3: Yangzhou, Huzhou, Taizhou) were randomly selected. The study population comprised interns from the following hospitals: Shanghai Yangpu District Hospital, Nanjing Jiangning Hospital, The First Affiliated Hospital of Zhejiang University, Hangzhou Third People’s Hospital, Suzhou Wujiang District First Hospital, Ningbo Yinzhou District Hospital, Xuzhou Mining Group General Hospital, Shaoxing People’s Hospital, Yangzhou First People’s Hospital, Huzhou Central Hospital, Taizhou Central Hospital.

Inclusion Criteria: (1) Age ≥20 years; (2) Voluntary participation; (3) Active undergraduate clinical medicine interns currently undertaking hospital rotations.

Exclusion Criteria: Responses exhibiting logical inconsistencies, excessively short completion times (<2 min), extreme response patterns, invalid answers to open-ended questions, or duplicate submissions.

Sample size determination followed psychometric standards recommending 10–30 participants per scale item (26). For the 7 core Likert-scale items, this yielded a minimum target of 210 participants. We recruited 324 interns through convenience sampling, of whom 21 were excluded based on predefined criteria. The final analytical sample of 303 participants substantially exceeded the psychometric target, thereby providing robust statistical power for factor analysis and minimizing the risks of model overfitting and factor instability.

### 2.4 Data analysis

Statistical analyses were performed using SPSS 25.0 and AMOS 26.0. All inferential tests employed *α* = 0.05 as the significance threshold. Descriptive statistics summarized participants’ overall characteristics, knowledge, and attitude scales. Qualitative data are described as frequencies and percentages; quantitative data are described as mean and Standard Deviation (SD). The scale items in Parts II and III underwent reliability and validity validation, employing Cronbach’s α coefficient to assess internal consistency, KMO measure and Bartlett’s test of sphericity for EFA, and AMOS 26.0 for Confirmatory Factor Analysis (CFA). Normality was assessed using the nonparametric Kolmogorov–Smirnov (K-S) test across gender, age, and city-tier subgroups. For normally distributed data, independent samples t-tests were employed; for non-normally distributed data, Mann–Whitney U tests (two-group comparisons) or Kruskal-Wallis tests (multi-group comparisons) (27)were utilized for mean comparisons. Spearman’s rank-order correlation analysis was performed to assess the relationship between knowledge and attitude dimensions. For Part III multiple-choice questions, multiple response analysis calculated option frequencies, with chi-square goodness-of-fit tests examining proportional differences across options and chi-square tests comparing distributions between subgroups. Part IV open-ended responses underwent a directed content analysis (28) using a predefined coding framework. Two doctoral researchers, independent of the study design, performed independent dual coding of all responses, a process totaling 25 h (mean: 5 min/response) across three consensus sessions over 10 days. This process achieved excellent inter-rater reliability*κ* = 0.82(κ > 0.80 represents almost perfect agreement beyond chance (29)). Coding discrepancies (14% of initial codes) were resolved systematically: consensus discussions resolved 85% of divergences, with unresolved cases arbitrated by the principal investigator using predefined decision rules. Final thematic frequencies were quantified to determine prevalence rates, supplemented with representative quotations to illustrate thematic essence while maintaining respondent anonymity.

### 2.5 Ethical and consent approval

This study was reviewed and approved by the Ethics Committee of Hangzhou Third People’s Hospital (Approval Number: 2025KA064). The committee granted a waiver of written informed consent as the research involved no more than minimal risk to participants, and the study utilized fully anonymized data collection with no personally identifiable information collected. In accordance with the ethical principles of the Declaration of Helsinki, comprehensive information regarding the research objectives, data handling procedures, and participant rights was prominently provided on the first page of the online questionnaire (Supplementary Material 1). Participants were explicitly informed that proceeding to and submitting the questionnaire would constitute their implied consent to participate in the study. This consent procedure was approved by the ethics committee.

## 3 Results

### 3.1 Sociodemographic characteristics of interns

CLKAQ questionnaires were distributed via the WeChat-based Questionnaire Star mini-program, yielding 324 submitted responses. After data screening excluding 21 invalid entries, 303 valid questionnaires were retained for analysis. The cohort comprised 75.2% interns aged 23–25 years and 24.8% aged 20–22 years. Gender distribution was 55.4% female and 44.6% male. Geographically, 45.2% represented tier-1 cities, 27.7% tier-2 cities, and 27.1% tier-3 cities.

Descriptive analysis of interns’ knowledge and attitude scales revealed distinct characteristics (Table 1). Based on 5-point Likert scoring, the 303 participants exhibited relatively low self-perceived knowledge scale scores (Q1-Q4; mean 2.22 ± 0.42), positioning in the lower-medium range. Conversely, attitude scale scores were notably higher (mean 4.05 ± 0.31), falling within the upper-medium range. Standard deviations for all items remained below 1 (range: 0.31–0.94), indicating low dispersion. Response distributions showed distinct clustering: knowledge items predominantly between Disagree and Neutral, while attitude items concentrated between Agree and Strongly Agree.

### 3.2 Scale outcomes by intern characteristics

Normality testing using K-S test across gender, age, and city-tier subgroups revealed non-normal distributions for all knowledge and attitude scale items (*p* < 0.001). Given the non-normality, the Mann–Whitney U test assessed differences between gender groups (male/female) and age cohorts (20–22/23–25 years), while the Kruskal-Wallis H test evaluated differences across city tiers (tier-1/tier-2/tier-3).

Comparative analysis of gender differences across scale items revealed statistically significant disparities on Q3, Q5, and Q7 (*p* < 0.05), with males demonstrating superior capabilities in independently interpreting laboratory reports (Q3: *Z* = −2.213), stronger endorsement of clinical laboratory knowledge’s critical role in clinical decision-making (Q5: *Z* = −5.267, *p* < 0.001), and heightened motivation to acquire additional knowledge (Q7: *Z* = −2.279) compared to female counterparts.

Analysis of age-group differences revealed statistically significant disparities on Q1, Q5, and Q7 (*p* < 0.05), with the 20–22 cohort demonstrating higher self-perceived adequacy of clinical laboratory knowledge for job requirements (Q1: *Z* = −3.153) compared to the 23–25 cohort, while the older group exhibited more positive attitudes regarding the critical role of laboratory knowledge in clinical decision-making (Q5: *Z* = −1.969) and stronger motivation to acquire additional knowledge (Q7: *Z* = −1.995).

Comparative Analysis Across City Tiers.

As shown in Table 4, interns from tier-3 cities exhibited significantly higher mean ranks than those in tier-1 and tier-2 cities across all knowledge (Q1-Q4) and attitude (Q5-Q7) scale items (*p* < 0.05), indicating stronger learning demands and more positive attitudes toward clinical laboratory medicine.

**Table 4:** Group differences in CLKAQ scale scores (nonparametric tests).

Dimension	Item	Gender (Mann–Whitney U)		Age Group (Mann–Whitney U)		City Tier (Kruskal-Wallis)		*Post Hoc*
		*Z*	*p*	*Z*	*p*	*H*	*p*	
Knowledge	Q1	−0.084	0.933	−**3.153**	**0.002**	91.863	<0.001	**a,b,c**
	Q2	−1.404	0.160	−1.891	0.059	85.726	<0.001	**a,b,c**
	Q3	−**2.123**	**0.034**	−1.179	0.238	103.930	<0.001	**a,b,c**
	Q4	−1.722	0.085	−1.559	0.119	97.206	<0.001	**a,b,c**
Attitudes	Q5	−**5.267**	**< 0.001**	−**1.969**	**0.049**	**18.068**	**< 0.001**	**a,c**
	Q6	−1.821	0.069	−0.245	0.806	28.850	<0.001	**b,c**
	Q7	−**2.279**	**0.023**	−**1.995**	**0.046**	7.414	0.025	–

Z: Mann–Whitney U test statistic (standard normal approximation), H: Kruskal-Wallis test statistic (chi-square distributed), *p*: Asymptotic *p*-value (two-tailed for gender/age; omnibus for city tier); Bolded *p*-values indicate statistical significance (*p* < 0.05); Gender: Male vs Female; Age Group: 20-22y vs 23-25y; Post Hoc Pairwise Comparisons (Dunn-Bonferroni test, *p* < 0.05): a: Tier-1 vs. Tier-2 significant, b: Tier-1 vs. Tier-3 significant, c: Tier-2 vs. Tier-3 significant.

### 3.3 Analysis of multiple-choice responses on clinical laboratory attitudes

Significant variations were observed across all multiple-choice factors (*p* < 0.001; Table 5). For knowledge preferences (Q8), interpretation of routine tests (Q8a) and clinical-diagnostic integration (Q8b) demonstrated the highest demand, followed by specialized laboratory disciplines (Q8e), while technical principles (Q8c) and quality control (Q8d) were less prioritized. Motivations for learning (Q9) showed that 98.1% of interns prioritized clinical decision-making enhancement (Q9a), career advancement (Q9c), and certification exam preparation (Q9b), with only 1.9% Response Rate (*n* = 17) citing Intrinsic interest (Q9d). Knowledge acquisition channels (Q10) were dominated by Internet resources (Q10f), Medical literature (Q10a), Hospital-based training (Q10b), and Academic lectures (Q10c) (collectively 88.3% Response Rate), whereas mentor guidance (Q10e) and online courses (Q10d) accounted for merely 11.8% Response Rate. Primary learning barriers (Q11) included theory-practice disconnect (Q11a) and lack of expert guidance (Q11d) (80.9% Response Rate), with secondary barriers—Insufficient resources (Q11c), time constraints (Q11b), and content complexity (Q11e)—comprising 19.2% (Full multiple-choice response data are available in Supplementary Table S1).

**Table 5:** Multiple-choice response analysis of preferences, motivations, channels, and barriers.

Core options (prevalence >20%)	Prevalence (%)	*X^2^*	*p*
Q8: Preferred knowledge domains			
Q8a: Routine test interpretation	97.4	276.452	**<0.001**
Q8b: Diagnostic integration	92.1		
Q8c: Technical principles	22.1		
Q8d: Quality control and error analysis in diagnostic testing	20.8		
Q8e: Specialized disciplines	74.6		
Q9: Primary motivations			
Q9a: Clinical decision-making	98.7	250.664	**<0.001**
Q9b: Exam preparation	92.4		
Q9c: Career development	93.7		
Q10: Knowledge acquisition channels			
Q10a: Medical literature	89.4	276.615	**<0.001**
Q10b: Hospital training	77.9		
Q10c: Academic lectures	59.1		
Q10e: Mentorship	28.7		
Q10f: Internet resources	96.0		
Q11: Perceived learning barriers			
Q11a: Theory-practice disconnect	89.4	458.500	**<0.001**
Q11d: Inadequate mentorship	38.6		

Prevalence (%) = Respondents selecting option/Total participants (*N* = 303) × 100%; Only options with >20% prevalence shown; Bolded *p*-values indicate statistical significance at *α* = 0.05.

**Figure 1:**
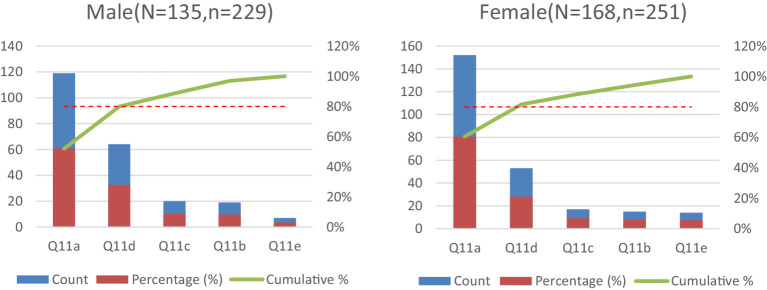
Pareto analysis of Q11a option distribution by gender. *N* = total participants; *n* = number of respondents selecting the option.

**Figure 2:**
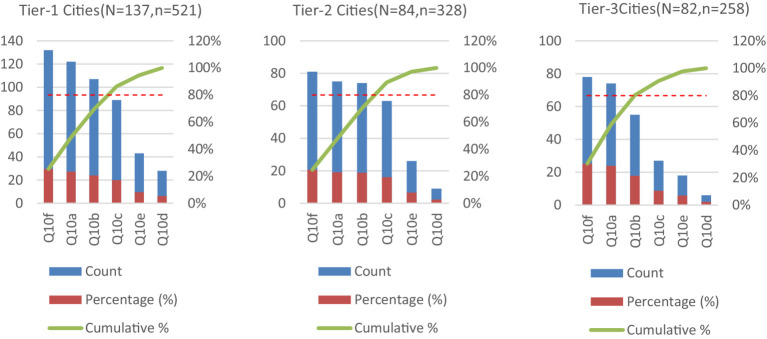
Pareto analysis of knowledge acquisition channel preferences distribution by city tier: Q10 item preferences. *N* = total participants; *n* = number of respondents selecting the option.

Chi-square analyses further identified (Statistical details for non-significant comparisons are provided in Supplementary Table S2): Gender-based disparities in perceived learning barriers (Q11: *χ*^2^ = 13.051, *p* = 0.023), City-tier variations in knowledge acquisition channels (Q10: *χ*^2^ = 55.872, *p* < 0.001). These patterns are visualized in Figures 1, 2. Q11a was a cross-gender core option (cumulative percentage >80%), selected by 119 males (88.1%) and 152 females (90.5%); males demonstrated a greater inclination toward Q11d, with 64 selections (27.95%) compared to females’ 53 (21.12%); males showed the lowest selection rate for Q11e (5.2%), while females had a higher rate for this option (8.3%). Regarding cumulative coverage comparison: the top two options (Q11a + Q11d) accounted for 79.92% of male responses, with males selecting approximately 1.7 options on average; for females, the top two options accounted for 81.68%, with females selecting approximately 1.5 options on average (251 choices among 168 females), indicating a greater tendency among males to select multiple options.

Across all city tiers, Q10f was the most prevalent option, selected by 132 individuals (96.35%) in tier-1 cities, 81 (96.43%) in tier-2 cities, and 78 (95.12%) in tier-3 cities. Preference variations between tiers emerged: tier-3 cities exhibited more concentrated selections (the top two options accounting for 58.91%), whereas tier-1 cities showed a relatively balanced distribution (the top two options accounting for 48.76%); Q10a displayed an ascending acceptance gradient from tier-1 to tier-3 cities (from 23.42 to 28.68%), indicating a potentially greater focus on medical textbooks/literature in smaller cities; Q10d remained the option with the lowest proportion across all city tiers. According to the significance under *Pareto Principle* (30), the top three options covered 69.30% in tier-1 cities, 70.13% in tier-2 cities, and 80.23% in tier-3 cities — confirming that options (Q10f/Q10a/Q10b) satisfy the majority of interns.

### 3.4 Open-ended responses regarding attitudes toward testing among undergraduate clinical medicine interns

All 303 interns (100%) endorsed the need for enhanced clinical laboratory medicine education (Q12). Subsequent qualitative analysis of Q13 yielded detailed suggestions for improvement. Directed content analysis of these responses generated 65 distinct codes, which were iteratively synthesized into 5 overarching themes (Mandatory Clinical Laboratory Rotations, Test Report Interpretation Workshops, Hospital Academic Lectures on Emerging Technologies, Monthly Microlearning Online Modules and Case-Based Diagnostic Sessions) outlining proposed training strategies. The identified themes, presented in Table 6, encompass a range of practical and formalized training approaches, reflecting interns’ preferences for diverse and structured educational formats. The most prevalent recommendation was for mandatory clinical laboratory rotations (27.4%), highlighting a pressing need to gain a better understanding of laboratory operations and thereby acquire more knowledge about laboratory testing. These suggestions provide a clear framework for refining laboratory medicine training during clinical clerkships (Table 6).

**Table 6:** Themes from qualitative analysis of interns’ suggestions (Q13) for improving clinical laboratory medicine training.

Theme	*n*	Prevalence *n* (%)	Representative Quote(s)
1. Mandatory Clinical Laboratory Rotations	83	27.4%	“A compulsory rotation of at least 2 weeks in the lab department is essential to understand the entire process and quality control.” “We need hands-on experience in specialized fields like molecular biology to see how it’s done in real practice.”
2. Test Report Interpretation Workshops	64	21.1%	“Regular workshops led by lab physicians would help us bridge the gap between theory and practice in interpreting reports.” “Quarterly case-based sessions on how to read CBC, coagulation, and biochemistry panels would be very beneficial.”
3. Hospital Academic Lectures on Emerging Technologies	63	20.1%	“Invite experts to give lectures on new technologies like molecular diagnostics and their clinical applications.” “We need to learn about the principles and limitations of new lab techniques through quarterly technical seminars.”
4. Monthly Microlearning Online Modules	60	19.8%	“Short, focused online modules (<1 h) each month on specific tests would be easier to fit into our busy schedule.” “Bite-sized learning content that we can access on our phones would be very efficient.”
5. Case-Based Diagnostic Sessions	33	10.9%	“Learning through real, anonymized patient cases where we have to select and interpret tests would develop our clinical reasoning.” “Simulated diagnostic scenarios using real patient data would make the learning stick.”

*n* = number of interns whose responses were coded into this theme; % = percentage of the total sample (*N* = 303). The sum of n exceeds 303 and percentages exceed 100% as responses could be coded into multiple themes.

## 4 Discussion

This study aimed to assess the self-perceived knowledge and attitudes toward clinical laboratory medicine among undergraduate medical interns in China, a population and topic that has not been comprehensively evaluated previously. Our findings reveal a significant discrepancy between interns’ modest self-assessed knowledge and their highly positive learning attitudes, alongside identifying specific gaps influenced by gender, age, and city tier. Furthermore, the study delineates preferred learning modalities and perceived barriers, providing a evidence-based foundation for targeted educational interventions. Since Dr. Gottfried’s (31) survey on establishing Laboratory Medicine courses in medical schools and publications by Smith (32) and Dr. Wilson (33) emphasized the significance of Laboratory Medicine knowledge, international attention to such curricula has gradually increased. Subsequently, Saffar et al. (10) designed an assessment of undergraduate medical students’ knowledge in Laboratory Medicine; however, this study had a relatively small sample size of 37 interns and employed a non-scale methodology. Professor Alosaimi (11) at Kingdom of Saudi Arabia and Bengayed et al. (34) in Tunisia, respectively, developed knowledge assessment questionnaires targeting specialized domains within Clinical Laboratory Medicine, aiming to underscore the critical importance of such knowledge. No study has comprehensively assessed self-perceived knowledge-attitude discrepancies among Chinese clinical interns. Building upon this foundation, we analyze the current challenges through our self-developed questionnaire (CLKAQ) and propose evidence-based improvements.

### 4.1 Knowledge-attitude discrepancy

This study identified a significant discrepancy between interns’ self-perceived knowledge of clinical laboratory medicine (mean score 2.22/5) and their proactive learning attitudes (mean score 4.05/5). This pattern reflects a hierarchical competence gap within Miller’s clinical assessment framework (35): while interns demonstrated strong motivation (*action level*), their operational capability in core competencies—exemplified by the low score in independently interpreting laboratory reports (Q3: 2.22; *shows how level*)—remained critically underdeveloped. Notably, Q3 recorded the lowest score (2.22), with only 20.8% of interns expressing confidence in interpreting basic reports. This highlights a critical deficit relative to the ACGME’s mandated competency of integrating laboratory results into clinical decision-making (1).

The predominant barriers to learning were a “curriculum-practice disconnect” (Q11a, 89.4%) and a “lack of expert guidance” (Q11d, 38.6%), underscoring a structural misalignment between laboratory medicine instruction and clinical practice requirements. Although a statistically significant positive correlation emerged between knowledge and attitudes (*r* = 0.171, *p* = 0.003), its weak magnitude suggests limited predictive utility of attitudes for knowledge levels. Nevertheless, the coexistence of a persistent knowledge gap and highly positive attitudes independently justifies targeted educational interventions.

This finding aligns with global studies reporting inadequate laboratory knowledge among medical trainees. For instance, Saffar et al. (10) found that 47.9% of Iranian medical students self-rated as ‘weak’ in test interpretation, consistent with our participants’ low confidence. Similarly, Alosaimi (11) reported that only 18.3% of Saudi interns passed basic microbiology assessments, reinforcing the widespread nature of this challenge. However, our study adds a crucial dimension by quantifying the acute awareness of this deficiency among Chinese interns, as evidenced by an overwhelming desire for enhanced knowledge acquisition (Q7, 97.7%)—an aspect not extensively detailed in previous international literature. The weak correlation further nuances this relationship, indicating that positive attitudes alone are insufficient to bridge knowledge gaps, necessitating structured educational reforms beyond mere awareness raising. Given the increasing complexity of laboratory testing, integrating practical laboratory medicine content into undergraduate medical curricula is more imperative than ever, a urgency underscored by Christian’s call for institutional reform (36).

### 4.2 Core issue of demand-resource mismatch

Responses to Q8 (“What types of clinical laboratory medicine knowledge do you most want to learn?”) reveal interns’ greatest needs lie in clinical-translational skills: Routine test interpretation (Q8a), Diagnostic integration (Q8b) and Specialized disciplines (Q8e). Conversely, Technical principles (Q8c) and Quality control and error analysis in diagnostic testing (Q8d) showed lower demand, indicating a need to reduce didactic instruction hours while increasing clinical scenario training. This aligns with the conclusions from Dr. Lawson’s (37) study: Laboratory sessions provide students opportunities to revisit concepts initially presented in traditional classroom settings and actively apply these concepts to case-based scenarios.

Responses to Q9 (“What are your primary motivations for learning clinical laboratory medicine?”) highlight practical drivers: enhancing Clinical decision-making (Q9a), Career development (Q9c), and Examination preparation (Q9b) – collectively emphasizing the applied utility of such knowledge. This strong external motivation underscores the perceived instrumental value of laboratory knowledge, a finding that corroborates the observations of Salinas et al. (38), who noted that since laboratory data informs 70% of clinical decisions, clinicians must be equipped to actively participate in these decision-making processes.

Analysis of Q10 (“How do you typically access clinical laboratory medicine knowledge?”) indicates interns primarily utilize Internet resources (Q10f, 96%), Medical literature (Q10a), Hospital-based training (Q10b), and Academic lectures (Q10c). Optimizing the quality and accessibility of these channels requires prioritized interventions: enhancing the authority and utility of Internet resources (Q10f) through clinical databases and guideline updates; integrating medical texts (Q10a) with digital platforms; and institutionalizing hospital training (Q10b) and academic lectures (Q10c). Notably, heavy reliance on unstructured learning via internet resources (Q10f) coexists with limited mentorship (Q10e, 28.7%). This self-directed paradigm exacerbates knowledge fragmentation, fundamentally undermining the core objective of “enhancing clinical judgment capabilities” (Q9a, 98.7%) and signaling critical resource misalignment. Consequently, elevating and formalizing the role of mentorship is imperative. This is strongly supported by evidence indicating that effective mentoring significantly enhances medical interns’ job satisfaction and aids in personal and professional career planning (39). Mentors should be equipped to deliver foundational medical knowledge more comprehensively, while medical education systems must innovatively leverage mentor expertise to create structured programs (40). Such structured mentorship not only enhances career selection confidence, increases scholarly output, and inspires careers in academic medicine (41), but also helps address challenges in mentor-mentee relationships, particularly for female interns, thereby demonstrably advancing equity across socioeconomic groups in medical training.

Analysis of Q11 (“What do you perceive as the main barriers to learning clinical laboratory medicine? “) identifies the foremost barrier as Theory-practice disconnect (Q11a), making curricular reform an immediate priority (as addressed in Section 4.1). Medical schools and residency programs should additionally incorporate interprofessional education, formal management training, and diversity-responsive teaching modalities (42). The secondary barrier—Inadequate expert mentorship (Q11d)—further underscores the critical significance of structured mentorship.

### 4.3 Educational implications of group disparities

Table 4 demonstrate that males exhibit greater confidence in report interpretation (Q3, *p* = 0.034), yet attitude scales indicate stronger learning motivation among females (Q7). This suggests males require enhanced training in knowledge rigor, while females benefit from increased practical confidence. Interns in tier-3 cities exhibit statistically significant higher mean rank values across knowledge and attitude scales compared to tier-1/2 cities, reflecting heightened engagement that may stem from primary healthcare settings’ heavier reliance on foundational testing. Notably, observed variation in responses to Q10 (“How do you typically access clinical laboratory medicine knowledge?”) across city tiers potentially stems from divergent learning environmental factors — a finding further supported by research emphasizing the practical implications of contextual learning influences, even in different trainee populations (43).

### 4.4 Synthesis of open-ended responses

The high utility of open-ended questions in uncovering respondent attitudes (44) is validated in this study, revealing undergraduate clinical interns’ critical demand for structured, specialized practical training in clinical laboratory medicine. The most endorsed recommendation was “Practical Rotation in the Testing Department” (27.4%), requiring ≥2-week immersive training to master key competencies including quality influencing factors and specialized fields like molecular biology. This reflects both the stringent operational standardization demanded in laboratory medicine (per CLSI EP23-A (45)) and the core need to enhance diagnostic accuracy through hands-on training – constituting a direct solution to the “knowledge-practice disconnect.” Complementary quarterly needs emerged for test report interpretation training (21.1%) and hospital academic lectures (20.1%). The former prioritizes laboratory physician-led interdisciplinary collaboration, addressing critical factors in translating laboratory data into clinical decisions. Diagnostic effectiveness fundamentally depends on two components: clinicians’ meticulous data analysis and recognition of laboratory medicine’s significance throughout healthcare processes. As David (9) notes, research on diagnostic errors remains critically inadequate—progress demands urgent methodological reform. The latter focuses on emerging technologies like molecular diagnostics. Together, they establish a longitudinal “theory-to-practice” training continuum. Notably, while online training (19.8%) is valued for flexibility, participants specified fragmented microlearning parameters (≤1 h monthly sessions), confirming trainee preference for approaches proven to enhance knowledge retention and reinforce long-term memory (46). Though case analysis received relatively lower endorsement (10.9%), its essential requirement for authentic patient data integration is designed to cultivate clinical correlation thinking.

### 4.5 Recommendations for educational improvement

Consistent with the study’s objective to inform curriculum optimization and educational strategy development, the following evidence-based recommendations are proposed based on the identified gaps in self-perceived knowledge, positive attitudes, and learning preferences.

Curriculum Restructuring with Dual-Track Laboratory-Clinical Integration: Implement longitudinal integration using clinical cases to sequence laboratory test selection → report interpretation → diagnostic refinement (e.g., quarterly case workshops per Q13 feedback). Embed mandatory laboratory rotations (27.4% support rate) within clinical clerkships, requiring ≥2-week placements emphasizing critical value management (Q4 mean score: 2.23) and hands-on experience with pre-analytical factors and basic instrumentation. This directly addresses the identified “knowledge-practice disconnect” (Q11a).

Resource Ecosystem Optimization: Develop authoritative, accessible knowledge repositories by curating and integrating high-quality Internet resources (Q10f, 96%) and medical texts (Q10a, 89.4%) with evidence-based guidelines (e.g., CLSI EP23-A). Establish structured mentor-trainee matching systems involving laboratory physicians to address the critical guidance deficiency (Q11d, 38.6%) through regular, laboratory-led report interpretation sessions (Q13, 21.1%). Effective mentoring is crucial not only in specialized residencies but also in undergraduate medical education to enhance confidence, clinical reasoning, and professional development.

Technology-Enabled Learning Innovation: The overwhelming reliance on Internet resources (Q10f, 96%) for self-directed learning, coupled with the identified barriers of “theory-practice disconnect” (Q11a) and “lack of expert guidance” (Q11d), underscores a critical strategic imperative: to transform fragmented information consumption into structured, effective education. We propose a multi-faceted digital strategy: First, develop AI-powered, adaptive learning platforms that curate authoritative information and provide personalized, on-demand guidance, effectively simulating the expert mentorship that is physically lacking. This directly addresses the accessibility and guidance gaps. Second, institutional investment in high-quality, gamified interactive modules is crucial. Evidence demonstrates that such modules significantly improve knowledge retention, engagement, and motivation in laboratory medicine education (16). These tools can bridge the theory-practice gap by immersing students in virtual clinical scenarios for test selection and interpretation.

## 5 Limitations and future directions

The study’s exclusive focus on hospitals in China may limit generalizability. Future research should validate the questionnaire’s external validity through multi-regional sampling. Uncontrolled confounding factors (e.g., internship duration, rotation departments) warrant stratified analysis or covariate adjustment in subsequent study designs. Beyond knowledge and attitudes, variables like career prospects, compensation structures, and professional development pathways may significantly influence learning outcomes; thus, their potential interaction effects should be investigated. A key limitation is the reliance on self-perceived knowledge rather than objective assessment of factual knowledge, which may introduce bias. Future studies should incorporate objective knowledge tests. Reliance solely on self-assessment of knowledge levels (without standardized assessments) necessitates future integration of objective knowledge evaluations to enhance the questionnaire’s real-world problem-reflecting capacity.

We anticipate that intensified focus on medical education reform and clinician training will drive continuous optimization of undergraduate clinical medical internships to address evolving educational demands.

## 6 Conclusion

This study provides the first comprehensive assessment of self-perceived knowledge and attitudes toward clinical laboratory medicine among undergraduate medical interns in China. The findings reveal a critical discrepancy between markedly positive learning attitudes (mean score 4.05/5) and deficient self-assessed knowledge (mean score 2.22/5), particularly in core competencies like independent report interpretation. This knowledge-attitude gap, exacerbated by a pronounced theory-practice disconnect and inadequate mentorship, underscores a systemic vulnerability in current clinical training paradigms.

The implications of these findings extend beyond identifying a problem; they offer a data-driven blueprint for educational reform. The strong, expressed demand for practical, immersive training—quantified through both quantitative and qualitative data—mandates a shift from theoretical didactics to integrated, experiential learning. Our evidence-based recommendations, including mandatory laboratory rotations, structured mentorship programs, and technology-enabled learning innovations, provide concrete pathways to achieve this shift. By addressing these specific gaps, medical educators can directly enhance interns’ diagnostic reasoning capabilities, ultimately contributing to improved patient safety and reduction in diagnostic errors.

The CLKAQ, rigorously developed and validated in this study, serves as a reliable tool for ongoing evaluation and benchmarking in this field. While the study’s focus on Eastern China may limit immediate generalizability, the methodological framework and identified themes offer valuable insights for other medical education systems facing similar challenges.

Future research should prioritize multi-institutional and longitudinal studies to validate these findings across diverse regions and to assess the long-term impact of the proposed interventions. Integrating objective knowledge assessments with self-perceived measures will further refine our understanding of these competencies.

In summary, this research confirms that empowering future physicians with robust laboratory medicine skills requires more than motivation—it demands structural educational change. By implementing the proposed strategies, medical education systems can bridge the identified chasm between enthusiasm and competency, thereby preparing a more clinically proficient generation of physicians.

## Data Availability

The original contributions presented in the study are included in the article/Supplementary material, further inquiries can be directed to the corresponding author.
