# Supplementary material for: Knowledge and attitudes toward clinical laboratory medicine among undergraduate medical interns in China: a cross-sectional survey
Source: Front Med (Lausanne). 2025 Oct 13;12:1671631. doi: 10.3389/fmed.2025.1671631 (PMC12554674; doi:10.3389/fmed.2025.1671631)
Supplement: Supplementary file 1 [file Table_1.docx]

Supplementary Material

# Supplementary Data

## Raw data:

## 0624C(Raw data).xls

## Clinical Laboratory Knowledge and Attitudes Questionnaire

Questionnaire.doc

## content validity of the questionnaire

Focus group ratings on content validity of the questionnaire.xls

# Supplementary Figures and Tables

## Supplementary Tables (S1-S2, This content is not displayed in the main article but is provided as ​Supplementary Material for interested readers to access.)

| Table S1 Multiple-Choice Response Analysis | | | | | | |
| --- | --- | --- | --- | --- | --- | --- |
| Item | Option | Freq. | Response Rate (%) | Prevalence Rate (%) | 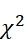 | p |
| Q8 | Q8a Interpretation of routine laboratory tests | 295 | 31.7 | 97.4 | 276.452 | **<0.001** |
|  | Q8b Interpretation of test results in the context of clinical diagnosis | 279 | 30.0 | 92.1 |  |  |
|  | Q8c Technical principles and operational procedures of laboratory assays | 67 | 7.2 | 22.1 |  |  |
|  | Q8d Quality control and error analysis in diagnostic testing | 63 | 6.8 | 20.8 |  |  |
|  | Q8e Specialized laboratory disciplines | 226 | 24.3 | 74.6 |  |  |
|  | Total | 930 | 100.0 | 306.9 |  |  |
| Q9 | Q9a Enhancing Clinical Decision-Making Skills | 299 | 34.0 | 98.7 | 250.664 | **<0.001** |
|  | Q9b Certification/licensing examinations | 280 | 31.8 | 92.4 |  |  |
|  | Q9c Professional development requirements | 284 | 32.3 | 93.7 |  |  |
|  | Q9d Intrinsic motivation for skill mastery | 17 | 1.9 | 5.6 |  |  |
|  | Total | 880 | 100.0 | 290.4 |  |  |
| Q10 | Q10a Medical textbooks/literature | 271 | 24.5 | 89.4 | 276.615 | **<0.001** |
|  | Q10b Hospital-based training | 236 | 21.3 | 77.9 |  |  |
|  | Q10c Academic lectures | 179 | 16.2 | 59.1 |  |  |
|  | Q10d Online courses | 43 | 3.9 | 14.2 |  |  |
|  | Q10e Mentorship | 87 | 7.9 | 28.7 |  |  |
|  | Q10f Internet resources | 291 | 26.3 | 96.0 |  |  |
|  | Total | 1107 | 100.0 | 365.3 |  |  |
| Q11 | Q11a Curriculum-clinical practice disconnect | 271 | 56.5 | 89.4 | 458.500 | **<0.001** |
|  | Q11b Time constraints | 34 | 7.1 | 11.2 |  |  |
|  | Q11c Insufficient learning resources | 37 | 7.7 | 12.2 |  |  |
|  | Q11d Inadequate expert mentorship | 117 | 24.4 | 38.6 |  |  |
|  | Q11e Excessive content complexity | 21 | 4.4 | 6.9 |  |  |
|  | Total | 480 | 100.0 | 158.4 |  |  |
| *Note:​ Bolded p-values indicate statistical significance at α = 0.05; Response Rate (%)​​ = Frequency of option selected / Total responses × 100%(Indicates relative popularity among choices);​Prevalence Rate (%)​​ = Respondents selecting option / Total participants (N=303) × 100%(Reflects overall adoption level),Prevalence rates may exceed 100% as multiple responses were allowed per participant.* | | | | | | |

| Table S2 Chi-Square Analysis of Group Differences in Multiple-Choice Responses | | | | | | |
| --- | --- | --- | --- | --- | --- | --- |
| Item | Gender | | Age | | City Tier | |
|  | 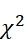 | P | 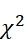 | P | 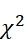 | P |
| Q8 | 3.573 | 0.612 | 2.853 | 0.723 | 9.534 | 0.482 |
| Q9 | 5.563 | 0.234 | 3.390 | 0.495 | 9.053 | 0.338 |
| Q10 | 3.157 | 0.789 | 1.789 | 0.938 | 55.872 | **<0.001** |
| Q11 | 13.051 | **0.023** | 8.412 | 0.135 | 9.512 | 0.484 |
| *Note: Bolded p-values indicate statistical significance at α = 0.05 (two-tailed)* | | | | | | |
